# Antibody-Oligonucleotide Conjugates: A Twist to Antibody-Drug Conjugates

**DOI:** 10.3390/jcm10040838

**Published:** 2021-02-18

**Authors:** Julien Dugal-Tessier, Srinath Thirumalairajan, Nareshkumar Jain

**Affiliations:** 1 NJ Bio, 675 US Highway 1, Suite B129, North Brunswick, NJ 08902, USA; naresh.jain@njbio.com; 2 Seagen, 21717 30th Drive, S.E., Bothell, WA 98021, USA; sri@seagen.com

**Keywords:** antibody-oligonucleotide conjugates (AOCs), oligonucleotide, siRNA, precision medicine

## Abstract

A summary of the key technological advancements in the preparation of antibody–oligonucleotide conjugates (AOCs) and the distinct advantages and disadvantages of AOCs as novel therapeutics are presented. The merits and demerits of the different approaches to conjugating oligonucleotides to antibodies, antibody fragments or other proteins, mainly from the perspective of AOC purification and analytical characterizations, are assessed. The lessons learned from in vitro and in vivo studies, especially the findings related to silencing, trafficking, and cytotoxicity of the conjugates, are also summarized.

## 1. Introduction

Antibody-drug conjugates (ADCs) have evolved to be an important new arsenal in the treatment of various cancers due to their preferential delivery of chemotherapeutics to cancer cells [1]. This approach has allowed an improvement of the therapeutic index of many antibodies. It has similarly aided the utilization of many small molecules that are potent and efficacious but possess properties that preclude them from being developed into a chemotherapeutic. For example, well-known potent payloads such as calicheamycins, auristatins and maytansinoids were found to be too toxic to be used as standalone single-agent chemotherapeutics but were found to have favorable clinical benefits in liquid (cancers of the blood such as leukemias, lymphomas and myelomas) and solid tumors when conjugated to antibodies [2,3]. Antibodies have proven to be ideal delivery agents, in general, due to their high specificity, long half-life and low immunogenicity [2]. The field of ADCs has evolved rapidly in the last decade, resulting in a better understanding of the role of target selection, release mechanism, payload potency and the role of each component in the overall activity profile. As more clinical data have become available, dosing levels and toxicity management of ADCs are better understood, leading to an increase in clinical success [3].

Although selectivity in an ADC is achieved through the antibody, non-specific toxicity is still observed. If more precise payloads could be used, the synergistic combination could potentially result in a more selective and safer ADC. Oligonucleotides qualify as such a class of precise payloads due to their ability to arrest protein production by homing in on specific genes. The use of small interfering RNA (siRNA) and anti-sense oligonucleotides (ASO) has rapidly evolved over the past decade [4]. Even though oligonucleotides offer selectivity, they suffer from challenges such as short serum stability, low membrane permeability, and lack of tissue selectivity. Antibodies, with their longer half-life, ability to selectively deliver therapeutics inside the cells, and targeting properties, make them ideal partners for the targeted delivery of oligonucleotides. ADCs suffer from systemic toxicity due to non-selective payloads, but the selectivity of oligonucleotides could enhance the ability of conjugates to only affect target disease cells. Antibody–oligonucleotide conjugates (AOCs) combine the high precision of siRNA and ASOs with the deliverability of antibodies, thus synergizing the advantages of both technologies.

The field of AOCs started initially as a means to develop powerful diagnostic tools but has evolved more recently as a targeted therapeutic approach for many diseases [5]. The use of AOCs grew rapidly due to their ability to detect antigens with greater sensitivity with PCR compared to fluorescent or other colorimetric methods [5]. As the field of oligonucleotides matured and selective tissue delivery became an important challenge for clinical use, AOCs transformed into potential single-agent therapeutics. Both the ADC and the oligonucleotide field have had tremendous growth in chemistry, conjugation and analytics over the past decade, which has increased the chances of generating a successful AOC. 

We will highlight the development and use of AOCs for the potential treatment of diseases. The focus will be on conjugation methods and the in vitro and in vivo data generated from these AOCs. The review will point out differences and similarities between ADCs and AOCs. While some of the aspects of development and preparation of ADCs and AOCs might seem related, they are different modalities with their own unique challenges.

## 2. Conjugation of Oligonucleotides to Antibodies

**Figure 1:**
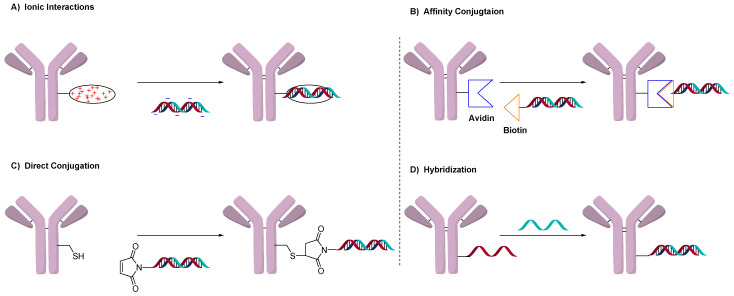
General methods to conjugate oligonucleotides by (**A**) electrostatic interactions, (**B**) affinity between biotin and avidin, (**C**) directly to antibody and (**D**) using double-strand hybridization.

Conjugation of drug-linkers in ADCs exclusively uses the direct conjugation method. Oligonucleotides, on the other hand, have more conjugation methods than typical small molecules. Figure 1 illustrates the four general methods used to prepare AOCs. These methods involve ionic interactions (Figure 1A), affinity binding (Figure 1B), direct conjugation (1C) and utilization of the double-strand as a conjugation moiety (Figure 1D). The use and advantages of each will be discussed.

Conjugating oligonucleotides to proteins generates different challenges from the more traditional conjugation of small molecules. Challenges in the preparation of ADCs include heterogeneity, hydrophobicity of the drug-linker, aggregation and stability of the linker. The size of the drug and its charge contribution to the resultant conjugate also have a significant impact on the preparation of an ADC, but these challenges are amplified in AOCs. For example, the molecular weight (MW) of a typical drug-linker in an ADC is less than 2 kDa, while an oligonucleotide is larger and can have an MW greater than 10 kDa. This means that the influence of the oligonucleotide on the physical and chemical properties of the conjugated antibody (150 kDa) will be larger than for small molecules. Thus, the analytical and purification methods used in the preparation of ADCs may not translate to AOCs.

Oligonucleotides with their negatively charged phosphate backbones are water-soluble, which can contrast with some small molecules found in ADCs. The negative charge that imparts solubility is also a challenge once conjugated to an antibody as it significantly changes the overall charge of the antibody. Conventional drug-linkers are usually neutral or have fewer than two charges/drug-linker and are optimized to not significantly change the properties of the parent antibody once conjugated. Even if the ADC has measurable differences by imaged capillary isoelectric focusing (icIEF) and ion-exchange (IEX) chromatography compared with the antibody, the resultant conjugate behaves much like the parent antibody and can be analyzed and purified as such. However, oligonucleotides are inherently negatively charged (with over 20 negative charges) and will dominate the overall charge profile of the AOC. Thus, the analysis and characterization of an AOC is anything but conventional or straightforward. For example, while most mass-spectrometry (MS)-based methods used in the characterization of ADCs rely on detection using the positive mode, AOCs with their many negative charges are almost invisible under those detection conditions.

Another main difference in the characterization of ADCs and AOCs is how the drug/oligonucleotide-to-antibody ratio can be calculated. Typical standard drug to antibody ratio (DAR) characterization for ADCs involves reverse-phase HPLC and/or hydrophobic interaction chromatography (HIC), which rely on the hydrophobicity of the drug-linker. These chromatographic methods are not transferable to the determination of oligonucleotide to antibody ratios (OAR). Methods used to determine OAR will be discussed in the individual conjugation method sections.

With an ADC, the conjugate behaves much like an antibody and not like a small molecule; with an AOC, the conjugate behaves both like an antibody and an oligonucleotide. Because of such differences, most recent advances in the purification and analytics of ADCs will not transfer to AOCs. This creates new challenges and the need for novel approaches for the purification and characterization of AOCs.

### 2.1. Conjugation Using Ionic Interactions

The addition of linkable groups to oligonucleotides is not trivial and can result in the synthesis of uncommon or expensive nucleotides [6]. However, the negative charge of the oligonucleotide backbone presents an attractive avenue for the preparation of conjugates. To this end, initial fusion or modification of an antibody with a multi-cationic moiety such as protamine or polyarginine allows for a general conjugation process [7,8]. The negative charge of the oligonucleotide backbone and positive charge of the protamine binds the oligonucleotide and protein strongly via ionic interactions. This method is applicable to many types of oligonucleotides since it does not require any chemical modification. Protamine is the most used multi-cationic group since it is an endogenous protein found in sperm and has been used to delay the activity of insulin [9].

The advantage of this method is its simplicity and flexibility in allowing the conjugation of different oligonucleotide sequences and technology with one polycationic protein. This could be useful for evaluating different gene knockdowns with the same antibody and measuring their activity in cells. Another advantage of ionic conjugation is that once the oligonucleotide enters cells, the polycationic complex acts as a lysosomal escape agent. In the lysosome, the polycationic complex acts as a proton sponge, and chloride ions diffuse inside to compensate for the charge imbalance causing osmotic swelling, which results in a leakier membrane [10]. This lysosomal escape is important, since oligonucleotides are not very membrane-permeable, and this allows for more oligonucleotides to enter the cytosol [10]. However, the main drawback of this method is that the interactions are ionic and thus, reversible. The conjugate could potentially be unstable, especially under changing pH or salt concentration. Another drawback of ionic interactions is the difficulty in determining the OAR, which is usually accomplished by fluorescence or PCR amplification.

The first example of antibody-mediated delivery of siRNA was published in 2005 by the Liberman group [7]. In this work, they prepared an antibody-protamine fusion protein and used it to deliver siRNA against the HIV-1 capsid gene gag. Using a fluorescein isothiocyanate (FITC)-labeled oligonucleotide, it was observed that each protamine could bind around six siRNA molecules. The antibody fragment targeted the HIV-1 envelope, which resulted in the inhibition of HIV replication in only HIV-infected primary T-cells in vitro. Since there are no in vivo models for HIV, a cancer cell line (B16) was transfected with the HIV envelope, and siRNA inhibiting oncogenes such as c-myc, MDM2 and VEGF were prepared. The power of protamine conjugation was clearly exemplified, as three different constructs could be generated rapidly using this conjugation method. Using a fluorescent-labeled oligonucleotide, it was found that a trastuzumab-based AOC was stable, and the siRNA was selectively delivered to ErbB2-expressing cell lines.

Following up on this discovery, a subsequent report used a human protamine fusion protein to silence genes in cells. The approach was to use AOCs to transfect cell lines, which was otherwise difficult to accomplish. One target of interest was the lymphocyte-function-associated antigen-1 (LFA-1), where selected genes could be silenced or modified in primary lymphocytes, monocytes and dendritic cells [11]. The internalization capacity of antibodies allowed gene silencing in hard-to-transfect cells in vitro. The AOC was also able to direct siRNA in LFA-1 engrafted lungs of SCID mice as an in vivo proof of concept.

**Figure 2:**
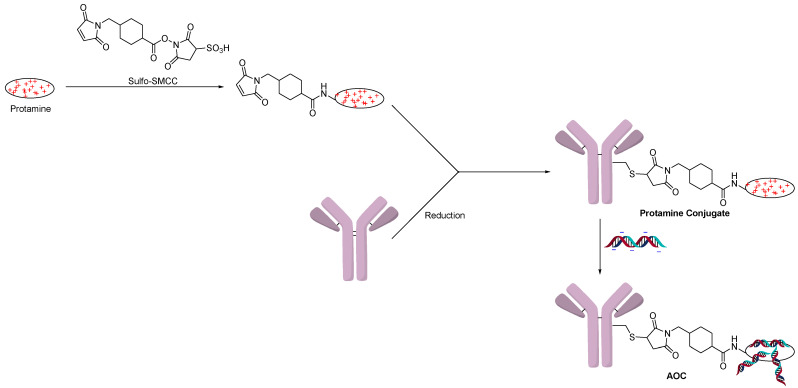
Protamine conjugated using Sulfo-SMCC.

A protamine anti-EGFR antibody was prepared by the Baumer group, who showed that an siRNA towards KRAS showed activity both in vitro and in vivo (Figure 2) [12]. Of note, KRAS has been a notoriously difficult oncogene to inhibit using standard small-molecule approaches [12]. The Baumer group compared protamine linked to both cysteine and lysine versus an antibody fusion construct. The activity was similar between the fusion antibody and the chemically modified ones, but the linker modified approach was more flexible [13].

Similarly, a SMCC-protamine antibody-siRNA conjugate was reported to inhibit TRIM24, which is overexpressed in prostate cancer [14]. An antibody towards prostate-specific membrane antigen (PSMA) was used to show selective internalization of FAM-siRNA [14]. The PSMA AOC showed potent activity in vitro and in vivo compared to negative controls.

**Figure 3:**
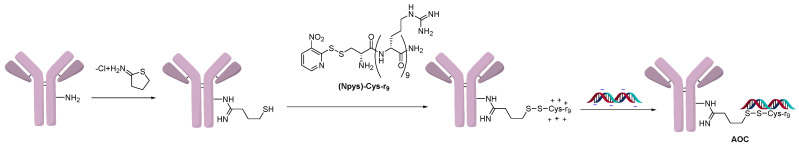
Polyarginine conjugation.

In another approach that utilized ionic interaction conjugation, lysine residues on an antibody were initially derivatized with Traut’s reagent and further elaborated into a disulfide polyarginine conjugation handhold (Figure 3). A STAT-3 siRNA sequence was delivered using an AOC targeting the Lewis-Y antigen (Figure 3) [15]. The polyarginine construct delivered siRNA specifically to cancer cells (A431) that overexpressed the Lewis-Y antigen and resulted in a 70% knockdown of STAT-3. In comparison, a conjugate in which the oligonucleotide was covalently linked to the antibody was also prepared using disulfide chemistry without polyarginine. Interestingly, the covalent conjugate was not efficacious on its own and required the addition of chloroquine, which promoted lysosomal escape. It was speculated that the superior activity of the polyarginine conjugate was because it helped the oligonucleotide escape the endosomes. This work demonstrates how ionic conjugation can benefit the activity of the AOC by promoting endosomal escape.

**Figure 4:**
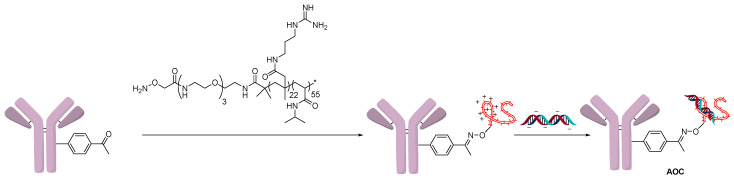
Polyarginine copolymer conjugation.

The Schultz group followed a similar strategy to create a polyarginine copolymer using RAFT polymerization (Figure 4) [16]. One end of the polymer contained a hydroxylamine, which formed an oxime bond by condensation with the non-natural amino acid para-acetylphenylalanine (pAF). The cationic polymer could then react with the negatively charged backbone of oligonucleotides. In this example, an FITC-labeled oligonucleotide was used to show targeted delivery to cancer cells using a trastuzumab-modified antibody. The knockdown activity of the AOC was measured by a PCR assay of mRNA levels of GAPDH, the siRNA target.

Using electrostatic interactions to conjugate oligonucleotides is an ingenious and generalizable method that is flexible across different types of oligonucleotides. An interesting advantage of ionic conjugation is that the polycations help the oligonucleotide escape the endosomes or lysosomes. Characterization of these conjugates with respect to the number of oligonucleotides attached is usually accomplished by using PCR or a fluorescent-labeled oligonucleotide. However, more comprehensive studies are needed to gain further insight into the in vivo stability, rate of release and ADME properties.

### 2.2. Avidin-Based Conjugation

In a similar approach, AOCs have also been successfully generated using the strong interactions between a biotin-labeled oligonucleotide and avidin (Figure 1B) [17]. A conjugate generated using this technique showed gene inhibition which was observed by transfecting human 293 cells with a luciferase gene. An antibody to the human insulin receptor showed that maximal inhibition of 90% was achieved after 48 h, and no inhibition was observed after 7 days by measuring luciferase activity. The advantage of avidin-based conjugation is the in vivo stability of the resultant conjugates. While polycationic complexes can aggregate in vivo due to changes in saline concentration, biotin-complexes are much more resistant to such effects [18].

Another approach used neutravidin as a linkable group for antibodies [19]. A podocyte-directed sheep divalent IgG was linked with neutravidin through cysteine, and protamine was conjugated to the antibody using a biotin tag [19]. An AOC was prepared using electrostatic interactions as described in Section 2.1. This AOC had increased in vivo efficacy found only with the directed siRNA but not with a random siRNA control. The goal of the study was to show that the antibody could be used to knockdown genes selectively in specific tissues. The future value of this approach would be to knockdown genes in specific tissues without generating the systemic effects of a knockdown mice model. Since it is expensive and time-consuming to develop transgenic mice, delivery of siRNA using antibodies offers a more general, easier approach to model disease states.

Although this is an efficient and practical method to conjugate, it still involves chemical modification of the antibody and the oligonucleotide or protamine with either biotin or streptavidin. Even if the bond between streptavidin and biotin is strong and efficient, this is a hybrid method that involves steps as described in Section 2.1 as well as those in the direct conjugation method described in the next section. Although it offers a stable bond, this method of generating AOCs is not as general as protamine and usually requires as many steps as other conjugation methods.

### 2.3. Direct Conjugation

The direct conjugation method is more analogous to ADCs in which a linkable group is added to the oligonucleotide and conjugated directly to a lysine, cysteine, or an engineered amino acid of the antibody. This method allows the use of the same linker versatility found in ADCs such as cleavable, disulfide, and non-cleavable linkers [20]. This method also has the advantage that linkers are smaller and have less impact on the overall conjugate, compared to the larger protamine or avidin complexes. On the other hand, each oligonucleotide construct must contain a linker handhold and be chemically modified to attach the linker. The linker technology must be compatible and stable in the presence of DNA or RNA and their double-strand annealing process.

The linker in the siRNA is usually incorporated into the sense strand rather than the anti-sense strand. The reasoning behind selecting the sense strand is that the anti-sense strand is the one used in the RNA-induced silencing complex (RISC) that results in gene silencing [21]. Modification of the sense-strand removes the chances that the residual linker group or the modified nucleotide will interfere with the siRNA binding to RISC.

AOCs prepared by the direct conjugation method do not incorporate lysosomal escape agents compared to conjugates prepared using the ionic method discussed in Section 2.1. As a result, this could potentially lead to a slow escape of the oligonucleotide from the lysosome and thus, could impact the activity of the AOC.

**Figure 5:**
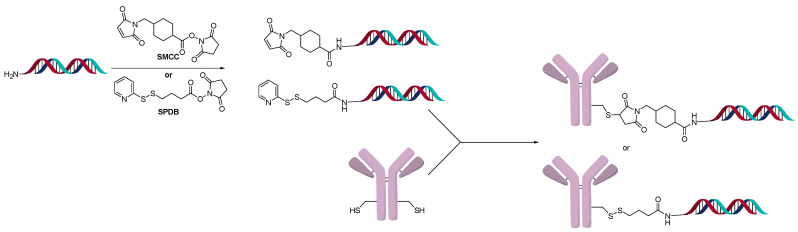
Direct conjugate method to oligonucleotide amine.

A powerful method to control DAR in ADCs is the introduction of an engineered site-specific cysteine on the antibody, which improves the overall properties of the ADC in vivo [22]. The site-specific cysteine engineered antibodies developed by Genentech are named Thiomab™. Analogous to ADCs, various Thiomab™ antibodies were conjugated to 21-mer oligonucleotides using SMCC and SPDB type linkers (Figure 5) [23]. The addition of the oligonucleotides added ~1/10th (for OAR =1) or ~1/6th (for OAR = 2) of the antibody weight and thus enabled the determination of OAR by MS. The AOCs were generated with oligonucleotides that contained modified nucleosides to improve serum stability. Using a fluorescent-labeled sequence, the internalization of the AOCs to the target cells was observed. Interestingly, only certain conjugates exhibited gene silencing activity, though they all internalized.

**Figure 6:**
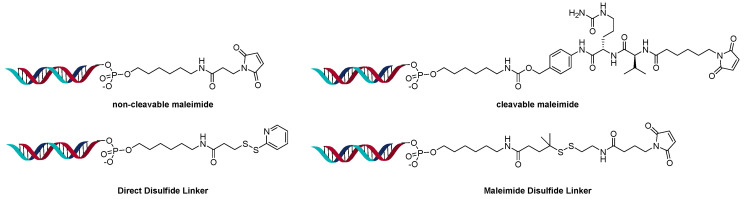
Conjugation through phosphate backbone.

Using the direct conjugation method with a variety of different linker types, the Sugo group was able to target skeletal and cardiac muscle with siRNA (Figure 6) [24]. The amount of siRNA present was determined using real-time polymerase chain reaction (RT-PCR). The stoichiometry of siRNA load (OAR) varied between 1.0 to 2.2 per Fab’.

**Figure 7:**
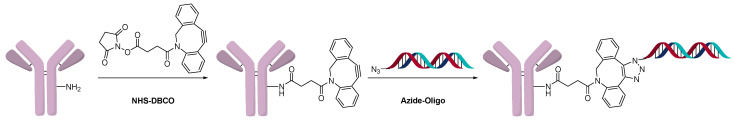
Azide-alkyne click chemistry based conjugation.

Another method of direct conjugation uses the DBCO-azide click or otherwise called the strain-promoted azide-alkyne cycloaddition (SPAAC) reaction to generate conjugates as shown in Figure 7. The advantages of this method are that one batch of antibody with the same linker-antibody ratio can be functionalized and different oligonucleotides can be conjugated orthogonally. Varying the equivalents of DBCO and/or azide modified oligonucleotide resulted in different higher-molecular-weight species (OAR), as expected. Fast protein liquid chromatography (FPLC) of Cy5-modified oligonucleotide was used to determine the average OAR, which was found to be 1.2 when four equivalents of the azide-functionalized oligonucleotide were used [25]. The reverse reaction in which the azide was conjugated to the antibody, and a DBCO-modified oligonucleotide was used, was also shown to be successful [26]. AOCs generated using click chemistry have used modified 2’-deoxy-2’-fluoro-β-d-arabinonucleic acid (FANA) oligonucleotides to generate more serum stable conjugates [27]. The click method of direct conjugation is often followed in the preparation of AOCs that are used in diagnostics [28]. Due to the orthogonality of click chemistry, and the compatibility with the oligonucleotide, this is a robust and popular method for conjugation. The hydrophobicity of DBCO and BCN reagents does not pose a challenge due to the water solubility of the oligonucleotide.

**Figure 8:**
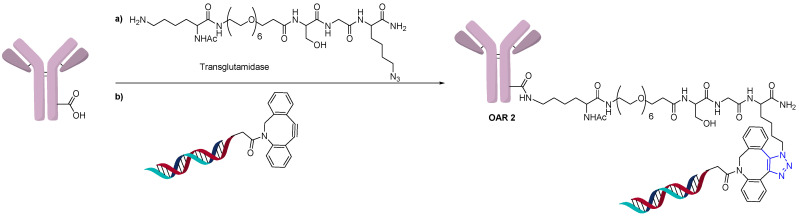
Transglutaminase-click chemistry.

Conjugation of oligonucleotides can also use the same site-specific conjugation technology used in the generation of homogeneous ADCs. Thus, an AOC with an OAR of 2 was prepared using site-specific conjugation by the addition of an azide-containing spacer and a transglutaminase compatible linker (Figure 8) [29]. The addition of a DBCO functionalized oligonucleotide could then undergo a click reaction to generate a homogeneous AOC with OAR of 2 by measuring MW shift by SDS-PAGE.

**Figure 9:**
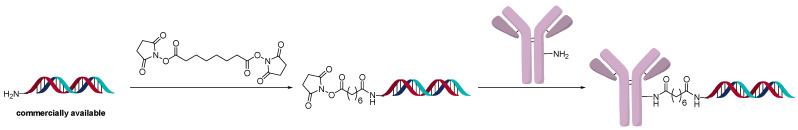
Disuccinimidyl linker.

Another interesting method for direct conjugation uses a disuccinimidyl linker (Figure 9). Even though the linker used is homobifunctional, when an excess of the linker is added to an amine-modified oligonucleotide, the major product is one linker/oligonucleotide. The intermediate linker-derivatized oligonucleotide is conjugated directly to the lysine of antibodies [6]. The modification of the antibody is determined by SDS-PAGE and MALDI-TOF. The main advantage of this method is that it is relatively inexpensive compared to other direct conjugation methods. Amine-containing DNA sequences are easier to access commercially than thio- or azide-modified sequences. The authors determined that it is the most cost-efficient and rapid method to generate AOCs and only requires 10 µg of antibody. This method was used to generate AOCs for use in diagnostics.

Using a non-natural pAF group, an AOC with trastuzumab was generated [30]. This AOC was used as a PCR primer to detect the presence of Her2+ samples with greater precision and sensitivity. This showcases the versatility of the orthogonal oxime linker chemistry as a protein labeling tool.

**Figure 10:**
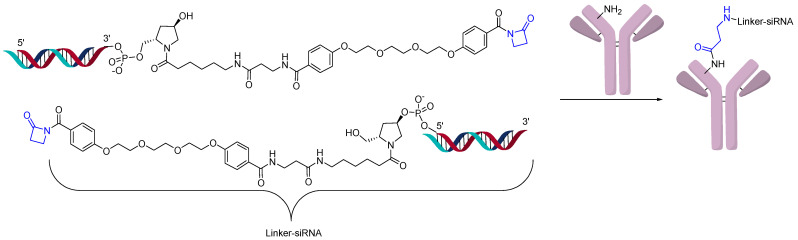
Beta-Lactam conjugation.

Recently, a site-specific lysine conjugation has been described in which a beta-lactam can react selectively to an H38C2 antibody as shown in Figure 10 [31]. This hidden lysine has a lower pKa (6.0) since it is in an accessible hydrophobic pocket, which in turn increases by orders of magnitude its reactivity towards electrophiles [32]. A targeting extra variable domain (Her2 or BCMA) is added to the H38C2 antibody, which generates the AOCs. The conjugation was achieved in good yields and resulted in a conjugate with an OAR of 2 using SDS-PAGE. Although no cytotoxicity was exhibited by this conjugate, silencing was observed in vitro.

Finally, non-engineered chemical site-specific conjugation technology has been recently reported using Fc affinity [33]. An advantage of this technology is that it generates homogeneous DAR using standard chemistries without the need for antibody engineering, thereby simplifying the analytics [34]. This approach was applied to AOCs and could help simplify the conjugation and analytics [35].

To summarize, the linker and antibody engineering used in ADCs can apply to AOCs when using the direct conjugation method. The direct conjugation method seems to offer the greatest flexibility, but judicious choice of linker compatibility and position on the oligonucleotide are still required. We expect that the direct conjugation will be a preferred method to prepare AOCs going forward.

### 2.4. DNA Origami or Hybridization Conjugation

**Figure 11:**
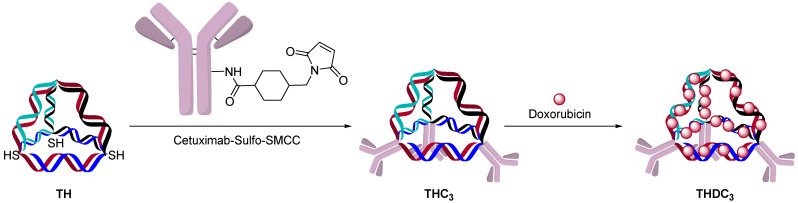
Doxorubicin intercalation into a 3D structures.

AOCs with double-strand oligonucleotides could be obtained by a hybridization approach. A single strand oligonucleotide is first conjugated to an antibody and a complementary strand is hybridized to form a double-strand AOC. The kinetics of double-strand hybridization is estimated to be >10^6^ M·s^-1^, which is orders of magnitude faster than click chemistry and other conjugation methods [36,37]. The rate and specificity of hybridization allows one to build defined structures and incorporate chemotherapeutics. One example was to use cetuximab as a targeting agent for the delivery of doxorubicin, which can be delivered to cells by intercalation into the oligonucleotide double-strand (Figure 11) [38]. This strategy allowed the incorporation of up to 480 molecules of doxorubicin/structure with three targeting antibodies.

**Figure 12:**
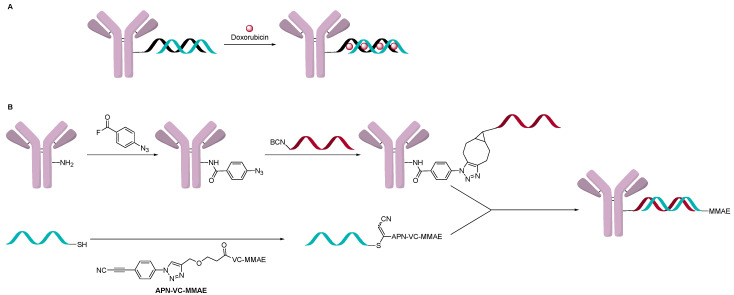
(**A**) Intercalation of doxorubicin and (**B**) hybridization of the drug-linker carrying strand.

This approach has been modified where the AOC is a carrier for a cytotoxin like an ADC (Figure 12A). A double-strand oligonucleotide was used to intercalate about eight doxorubicin per DNA molecule, which significantly increased the delivery of doxorubicin to cells [39]. In another instance, an AOC containing a single-strand oligonucleotide was formed using a direct attachment method, and separately, a complementary strain was attached with an ADC drug-linker and hybridized (Figure 12B). Two examples of this approach have been documented, and both have used trastuzumab as the targeting antibody [40,41]. In one example, the first oligonucleotide strand was conjugated to the antibody using interchain disulfides to give a variety of OAR, which was controlled by adjusting TCEP equivalents used during the reduction step. The complementary strain of DNA was modified with a thiol or an amine to conjugate MC-Val-Cit-PAB-MMAE, SMCC-DM1 or DM1 directly. AOCs with OAR ranging from 1.9 to 4.6 measured by SDS-PAGE and IEX chromatography were obtained and evaluated [40]. In vitro activity increased with OAR, and the activity observed was comparable to T-DM1. In another example, the single strand oligonucleotide was conjugated using a two-step click conjugation process through lysine [41]. The complementary strand was conjugated to MC-Val-Cit-PAB-MMAE by having a cysteine residue at the 3’ to add the drug-linker. The OAR distribution varied between 0 and 5 with an average of 1.9 measured by native MS. The in vitro assay data indicated selective activity towards target positive cells but was around 100-fold less potent than trastuzumab conjugated directly to the linker-payload (Val-Cit-PAB-MMAE). The author suggested that this could be due to the negative charge of the oligonucleotide, which can slow internationalization of the AOC.

The length of the oligonucleotide is very important for the hybridization approach. If the oligonucleotide sequence is too short, the duplex will not be stable enough in plasma, and if it is too long, it can form secondary structures [42]. In the case of intercalation with doxorubicin, the length of the sequence determines the amount of doxorubicin that can be carried by the AOC. Even though the kinetics are higher than for click chemistry, the cost of having a linker-payload attached to an oligonucleotide does not seem to favor conjugation through hybridization. This hybridization technique is very powerful for diagnostic use, but it seems more challenging for AOCs targeted towards disease.

### 2.5. Summary

All four methods of conjugation have yielded AOCs that could be characterized and tested in vitro and some in vivo. Most AOCs currently have OARs less than 4, but like ADCs, this could change over time. The overarching observation is that current methods used to determine OAR and distribution still pose a challenge for AOCs. The ability to attach a fluorescent probe to the oligonucleotide does allow measurement of OAR as an average, but without further separation of the species, their distribution is unknown. PCR is a powerful method to determine OAR by how much oligonucleotide is present, but distribution is also unknown. Due to the size and charge of the oligonucleotide, SDS-PAGE and IEX have been used to separate species and determine a distribution. Mass-spectrometry has been used for OAR determination, especially with homogeneous conjugation. General analytical methods for characterization are still a challenge, and as the field matures, these will surely become more routine.

## 3. In Vitro Data

This section will cover the use of in vitro data showing gene silencing, cytotoxicity, internalization kinetics, and processing of AOCs. If AOCs follow a similar pattern to ADCs, there could be little correlation between in vitro and in vivo results. Nonetheless, these in vitro studies can help shed some light on the nuances between conjugates. In vitro testing is necessary to understand parameters that play an important role in the in vivo activity of AOCs.

When it comes to in vitro mechanisms and controls, oligonucleotides do have some advantages compared to ADCs and other therapeutics. For example, a negative control can be made by just having a random oligonucleotide sequence of the same length and type. The addition of a fluorophore on the sense or anti-sense strand allows for the simultaneous tracking of the therapeutic and measurement of its activity with the same construct. In short, one well-designed AOC could give different levels of information, from cytotoxicity to stability and finally to imaging with one molecule.

### 3.1. Evaluating Gene Inhibition

The efficacy of an AOC will depend on its ability to inhibit protein production. The ability of the AOC to knockdown a gene can be performed by measuring mRNA [23], Western blotting or incorporating a green fluorescent protein (GFP) into the system. In combination with a fluorophore, the gene silencing activity and trafficking could be correlated together. A study by Xia et al. showed that by following luciferase activity, the highest-level gene silencing was observed at 48 h, and its silencing activity disappeared at day 7 [17]. Most studies measure gene silencing activity between 24 h and 72 h, since that is typically the time for maximal gene inhibition. At this time, it is not clear what minimum level of inhibition is needed for activity and if it correlates to in vivo data, but measuring gene inhibition appears to be important in vitro assay.

### 3.2. Internalization and Trafficking

**Figure 13:**
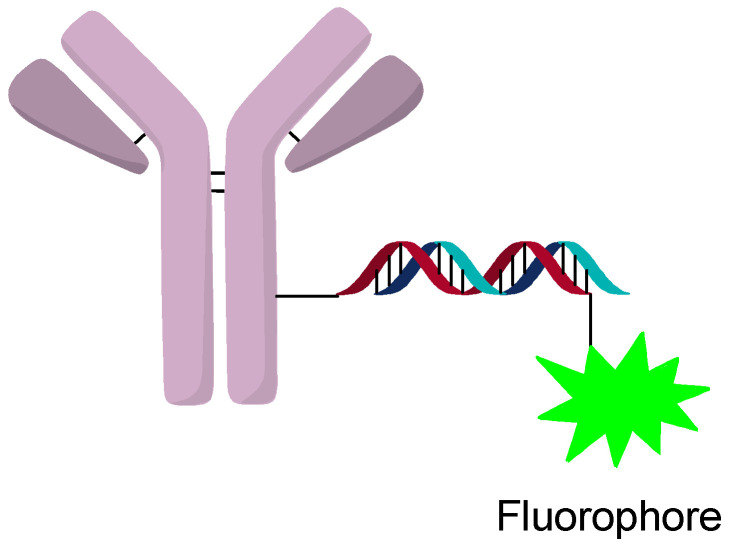
Fluorophore containing AOC.

The internalization and tracking of AOCs can be accomplished by labeling the oligonucleotide with a fluorophore or other tracking agent (Figure 13). Internalization of AOCs is generally observed for most targets, but escape from the lysosome seems more variable. Studies of different targets and silencing activity found that not all targets lead to silencing even when they are internalized [23]. Investigation by confocal microscopy showed strong co-localization of the AOC and LAMP2, which indicates processing through the endosomal pathway. It was postulated that differences in processing and/or escape from the lysosome can change the activity significantly between targets [23]. This reinforces the notion that some targets might require protamine or other lysosomal escape agents to increase the activity of the AOC. From in vitro studies, lysosomal escape seems to be an important consideration when developing an AOC and a major consideration in the selection of the target and construct design.

### 3.3. Cytotoxicity

Cytotoxicity is a useful measurement in oncology for the ability of the construct to arrest division and kill cells. Since AOCs are being investigated in genetic diseases as well as in oncology, cytotoxicity assays might not be the best measurement of activity. To put this into context, the IC_50_ of AOCs are generally in the nanomolar range, compared to ADCs, which are mostly in the picomolar range. This difference in activity could be due to the lack of lysosomal escape, trafficking differences or stability. The target of the oligonucleotide could also be important; some cells may be more sensitive to the silencing of a gene than others. No matter the reason, the selection of the antibody target will have to be matched with the activity of the AOC.

Cytotoxicity is an important measurement for AOCs that deliver cytotoxic agents. A doxorubicin intercalated cetuximab AOC showed 2.7 to 7 times more potent activity towards EGFR+ cell lines than free doxorubicin [39]. An in vitro cytotoxicity assay for a Her2 targeting oligonucleotide using DM1 and MMAE showed similar activity and specificity compared with conventional ADCs [40]. An experiment with a trastuzumab MMAE containing AOC showed single-digit nanomolar activity but was still about 10 times less potent than the comparable ADC [41]. In these two examples, the antibodies were different, but both targeted Her2-expressing cell lines and contained different cleavable MMAE oligonucleotide constructs. This illustrates that even when releasing the same MMAE molecule, with the same release mechanism, the oligonucleotide chemistry can impact the activity. Regardless, the activity of these oligo-conjugates is on par with early ADC work and remains promising.

### 3.4. Stability

The question of stability in blood is an important consideration due to the short half-life of oligonucleotides alone in plasma [41]. A study of DNA-based AOC tripled the half-life (5.7 days) compared with oligonucleotide alone (1.9 days) in human plasma [41]. A cetuximab oligonucleotide stabilized to protamine showed stability after 22 h in serum [12]. Early in vitro stability studies indicate that the oligonucleotide portion of the AOC does show enough stability to be dosed in vivo and have pharmacological activity.

### 3.5. Summary

Overall, the work presently published indicates that AOCs do offer a stability advantage over oligonucleotides alone, but more stable modified nucleotides are still necessary. AOCs seem to follow a similar internalization to the lysosome pathway as ADCs. A challenge for AOCs is the endosomal/lysosomal escape of the active oligonucleotide. AOC cannot simply rely on membrane permeability to enter the cytosol, unlike small molecules. The maximum gene silencing seems to occur between 24 h to 72 h in vitro. Current data show the ability of AOCs to inhibit cell growth by the delivery of either siRNA or cytotoxic agents. Overall, AOCs show promising results in vitro, but the concentrations required for activity are higher than their ADC counterparts. With promising in vitro data, the translation to in vivo models will be discussed next.

## 4. In Vivo Data

Conjugates have shown in vitro silencing, but without in vivo data, it is hard to know how much gene silencing is required for activity and if enough of the oligonucleotide can be delivered in xenograft models. AOCs have the same challenges as many other therapeutics in that even if silencing of protein is observed, it is unknown if that will lead to in vivo efficacy due to redundant systems in the cell or low levels of delivery. Thus, assessing the activity of the oligonucleotides alone is difficult. The selection of the antibody, the gene being silenced and the trafficking of targets will all be crucial for in vivo success.

### 4.1. In Vivo Imaging

The ability to add dyes to the oligonucleotide sense and anti-sense strands does allow for in vivo imaging of the AOC and oligonucleotide. Unfortunately, very few studies looking at animal models of the distribution of AOCs have been found.

A study performed using a Thiomab-based AOC towards TENB2 and silencing the PPIB gene was dosed 24 mg/kg three times over 3 days. The results showed 33% inhibition of the PPIB gene in mouse tumors [23]. An interesting note was that monitoring of a dye containing AOC showed that only the layers near the vasculature internalized the AOC. The authors speculate that the negative charge of the oligonucleotide repels the negative charge of the membrane, which could prevent penetration through a poorly vascularized tumor. These results also indicate the advantage of labeling the AOCs with a dye on the siRNA sequence to monitor location and activity.

The in vivo imaging is an important aspect to investigate further as early results indicate that tumor or tissue penetration is limited. The development of AOCs capable of distributing throughout the entire tumor or tissue could be key in their clinical success.

### 4.2. Efficacy

In general, the gold standard of preclinical data is efficacy in a relevant animal model. No matter how compelling or non-compelling the in vitro data is, programs that show poor in vivo efficacy usually do not progress into development. The limited in vivo data available on AOCs have shown mixed results and will be discussed in more detail below. The reasons for the mixed results are unknown and could include factors such as rescue pathway of inhibition and lack of potency or distribution. This does not mean that the concept of AOCs is flawed; some parameters will need to be optimized in future work.

The Lieberman group created an AOC with a cocktail of different gene-silencing siRNAs by ionic conjugation [7]. In mice implanted with a B16 engineered cell line overexpressing the HIV envelope, an siRNA cocktail to c-myc, VEGF and MDM2 showed in vivo activity. The AOC was dosed to deliver 3 × 80 micrograms of siRNA both intratumorally and intravenously on Days 0, 1 and 3, and the mice were sacrificed after day 9. The tumors were measured daily after 5 days, and at the end of the study, the tumors were collected and weighed. Both injection methods resulted in tumors weighing around 200 mg, while all the controls had weights around 800 mg. This does show the ability of ionic conjugation to deliver multiple siRNA sequences in one construct.

In an interesting approach, the Uckun group used bioinformatics to select a gene for inhibition in ALL. An AOC combining a CD19 targeting antibody and an anti-sense oligonucleotide to E2A-PBX1 was prepared using disulfide direct conjugation methodology [43]. The AOC was microdosed at 93 nmol/kg over 14 days in SCID mice. The mice treated with the AOC doubled their leukemia-free survival time compared to mice dosed with a control. Of note, the model used is resistant to radiotherapy, and this B-lineage ALL is challenging to treat. This a strong example that AOCs can be a general platform in which a gene can be identified, the sequence generated, and the construct tested in vivo without needing to find a small molecule inhibitor.

Based on similar reasoning, targets that are difficult to inhibit by small molecules could be rapidly inhibited by siRNA. An AOC using cetuximab and siRNA to KRAS was generated by ionic conjugation. This AOC showed significant tumor growth inhibition in three KRAS-mutated colon cancer cell lines when dosed at 4 mg/kg twice a week for 3 weeks. The activity was driven by the KRAS-inhibiting AOC as the cetuximab alone and cetuximab with a random oligonucleotide did not show any activity [12]. It is noteworthy that cell line HT29, which is also BRAF-mutated, was resistant to all treatment options. Histological investigation showed that the expression of the proliferation antigen Ki-67 was reduced only in KRAS-silencing groups. This research illustrates that difficult-to-drug targets could be treated using an AOC approach.

A research study on using a CD71-targeting AOC developed for the potential treatment of muscle diseases suggested that the AOC could be delivered selectively to the heart and calf muscle [24]. A running test for mice showed that silencing myostatin by intramuscular injection resulted in a major recovery of running performance. This approach indicates that AOCs could be used specifically for muscular disease and genetic disorders. Delivering oligonucleotides to specific tissues is an important goal that AOC could address.

An ASO against MXD3 conjugated to a CD22 antibody showed a significant survival time increase in a Reh xenograft AML model [26]. The animals were dosed twice a week for 3 weeks, and a dose-response could be observed from 0.2 mg/kg to 10 mg/kg. The free antibody was also co-dosed with the free ASO, and a dose response was observed but was significantly less effective than the AOC. Moving to patient-derived samples, a doubling of survival time was observed with doses as low as 0.2 mg/kg, and a greater survival time was noted for the 1 mg/kg dose. This ASO conjugate does look promising since it has a dose response and a clinically relevant dosing schedule.

A PSMA AOC that inhibited Trim24 was tested in vivo for efficacy [14]. At a dose of 5 mg/kg five times over 2 weeks, it showed tumor regression compared to a non-binding AOC and PBS controls. Interestingly, the negative random siRNA-control showed significant inhibition, but not as much as the active siRNA AOC. The author speculated that ADCC activity might be an important component of the observed activity, and the targeting PSMA antibody without siRNA was not reported. The role of the siRNA is still to be determined, but this is an encouraging result.

### 4.3. Pharmacokinetics (PK)

A study looking specifically at the stability and PK of AOCs has recently been published by the Rader group along with Alnylam. This study looked at the PK profile of the AOC and the naked mAb [31]. The sense strand of the oligonucleotide was biotinylated, which allowed the capture of the conjugated antibody with streptavidin. The antibody could be captured by standard ELISA methods, and thus, a PK profile of the conjugated and total mAb could be generated. It was reported that the half-life of the AOCs was 9.5 h and 12 h and that of the AOC antibody was 24 h and 37 h. The naked antibody had a half-life of 69 h. The shorter half-life could be due to cleavage of the phosphate bond between the linker and the oligonucleotide. The faster clearance of the total antibody in the AOC versus just the naked antibody could mean that the oligonucleotide also contributes to the removal of the bioconjugate faster due to its size or charge. Analogously, hydrophobic ADCs tend to clear faster [44]. As more PK data are published, a better understanding of the parameters leading to improved in vivo stability will help advance the AOC field.

### 4.4. Summary

Most papers with AOCs do show interesting in vitro data, but in vivo data are scarce. What has been published is promising. AOCs can silence multiple genes simultaneously in an in vivo setting. Using a bioinformatic approach, new targets can be identified and inhibited in difficult-to-treat models. Intracellular or tissue localization of AOCs can be monitored simultaneously using a dye incorporated within the oligonucleotide strand. In some cases, activity was observed with dosing below 1 mg/kg in xenograft models. There are still many unanswered in vivo questions for AOCs, especially around PK and in vivo stability of the oligonucleotides. Toxicology studies in mice, rats, and cynomolgus monkeys are also missing pieces of the AOC in vivo landscape. Toxicology studies will be published over time as more AOCs progress through preclinical development. In vivo data indicate that AOCs do have a strong potential to regulate gene expression and could be used as biotherapeutics, but much preclinical validation is required before they enter the clinic.

## 5. Conclusions and Outlook

The field of AOCs started as a diagnostic tool but over the last 10 years has emerged as a novel therapeutic modality. The field is in its infancy, and much more preclinical work is required. As of the writing of this review, we are not aware of any AOCs that have entered the clinic. On the other hand, siRNA conjugated to other targeting moieties have been approved [4]. Based on what has been published, major challenges in the field include analytical characterization, target and disease selection, trafficking and in vivo stability. As more tools become available, knowledge and mechanistic understanding of AOCs will evolve as well.

We envision that the landscape of AOCs will change rapidly over the next decade as the fields of ASO and siRNA advance; as new oligonucleotide constructs with improved stability, membrane permeability and lysosomal escape become available; as advances in linker technologies are refined for specific use in AOCs; as instruments and analytical methods are developed for easier measurement of metabolites, PK profiles and AOC characterization; and as target selection for AOCs that traffic directly to the cytosol are developed. These advances will significantly impact the next generation of AOCs. We speculate that with the large effort currently underway within many companies, the landscape will advance by leaps and bounds.

The interesting in vitro and in vivo results published appear to justify these large research efforts. AOCs lend themselves to a personalized approach where the oligonucleotide sequence could be tailored for select genetic defects in one tissue, cancer biopsies could lead to individual siRNA cocktails and proteins could be selectively inhibited. AOCs could become an important arsenal in precision medicine, and we look forward to the next 10 years of development.
